# Factors Influencing the Antibacterial Activity of Chitosan and Chitosan Modified by Functionalization

**DOI:** 10.3390/ijms22147449

**Published:** 2021-07-12

**Authors:** Cristina Ardean, Corneliu Mircea Davidescu, Nicoleta Sorina Nemeş, Adina Negrea, Mihaela Ciopec, Narcis Duteanu, Petru Negrea, Daniel Duda-Seiman, Virgil Musta

**Affiliations:** 1 Faculty of Industrial Chemistry and Environmental Engineering, Politehnica University of Timişoara, 2 Piata Victoriei, 300006 Timisoara, Romania; cristina.ardean@student.upt.ro (C.A.); adina.negrea@upt.ro (A.N.); mihaela.ciopec@upt.ro (M.C.); narcis.duteanu@upt.ro (N.D.); petru.negrea@upt.ro (P.N.); 2 Renewable Energy Research Institute-ICER, University Politehnica of Timisoara, 138 Gavril Musicescu Street, 300774 Timisoara, Romania; corneliu.davidescu@upt.ro; 3 University of Medicine and Pharmacy “Victor Babeș” Timișoara, 2 Piața Eftimie Murgu, 300041 Timișoara, Romania

**Keywords:** antibacterial effect, chitosan, chitosan derivative, functional groups

## Abstract

The biomedical and therapeutic importance of chitosan and chitosan derivatives is the subject of interdisciplinary research. In this analysis, we intended to consolidate some of the recent discoveries regarding the potential of chitosan and its derivatives to be used for biomedical and other purposes. Why chitosan? Because chitosan is a natural biopolymer that can be obtained from one of the most abundant polysaccharides in nature, which is chitin. Compared to other biopolymers, chitosan presents some advantages, such as accessibility, biocompatibility, biodegradability, and no toxicity, expressing significant antibacterial potential. In addition, through chemical processes, a high number of chitosan derivatives can be obtained with many possibilities for use. The presence of several types of functional groups in the structure of the polymer and the fact that it has cationic properties are determinant for the increased reactive properties of chitosan. We analyzed the intrinsic properties of chitosan in relation to its source: the molecular mass, the degree of deacetylation, and polymerization. We also studied the most important extrinsic factors responsible for different properties of chitosan, such as the type of bacteria on which chitosan is active. In addition, some chitosan derivatives obtained by functionalization and some complexes formed by chitosan with various metallic ions were studied. The present research can be extended in order to analyze many other factors than those mentioned. Further in this paper were discussed the most important factors that influence the antibacterial effect of chitosan and its derivatives. The aim was to demonstrate that the bactericidal effect of chitosan depends on a number of very complex factors, their knowledge being essential to explain the role of each of them for the bactericidal activity of this biopolymer.

## 1. Introduction

In 1859, Rouget discovered that by heating chitin in alkaline environment, a soluble material in organic acids can be obtained. The name of this material, “chitosan”, was given by Hoppe-Seyler in 1894, but the chemical structure was unknown until 1950 [1].

**Figure 1:**
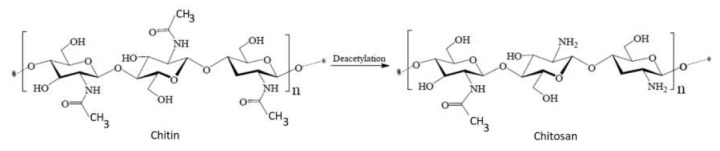
Partial deacetylation of chitin to chitosan according to [7].

Chitosan can be obtained by partial or total deacetylation of chitin, acetyl groups in the molecular chain of chitin being removed to form amino groups in chitosan. For this reason, chitosan can be classified as a copolymer composed mainly of 2-amino-2-deoxy-β-d-glucopyranose (glucosamine) and 2-acetamide-2-deoxy-β-d-glucopyranose units (N-acetylglucosamine) linked by glycosidic β(1→4) bonds [2,3,4,5,6] (Figure 1).

**Figure 2:**
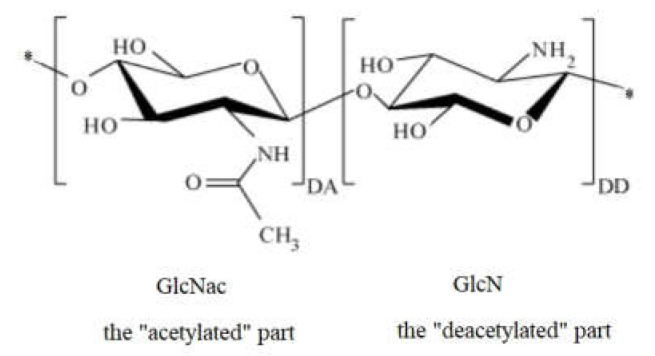
Molecular structure of the chitosan monomer repeated unit.

The structure of chitosan includes an “acetylated” part and a “deacetylated” part; the molecular structure of the monomeric units that are repeated in the chitosan molecule is shown in Figure 2. If the left side is 2-amino-2-deoxy-β-(1-4)-d-glucopyranose representing the degree of acetylation (DA) of chitosan, the right side is the deacetylated result of the left side, representing the degree of deacetylation of chitosan (DD).

**Figure 3:**
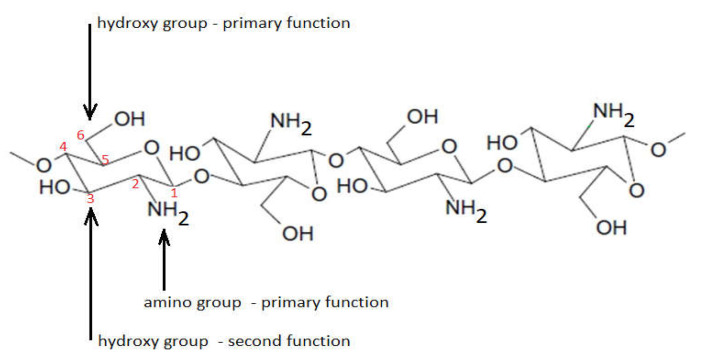
Linear structure of chitosan.

Monomer units are distributed randomly or as blocks around chitosan polymer [8,9,10]. This mode of distribution gives chitosan a rigid and uneven structure. The presence of hydroxyl groups located at C6 (hydroxyl group with main activity) and C3 (hydroxyl group with secondary activity) and amino groups (located on C2 of the molecule), which are highly reactive, with a concomitant tendency of intramolecular and intermolecular hydrogen bonds, results in the formation of the linear structure of the chitosan molecule (Figure 3).

**Figure 4:**
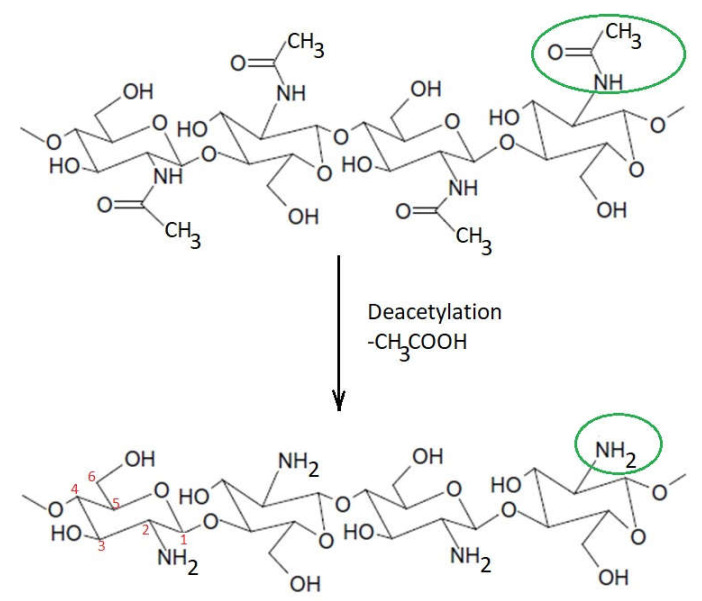
Difference between the structures of chitin and chitosan.

The major difference between chitin and chitosan consists of the amino groups (Figure 4). Thus, their physicochemical properties, which are correlated with their biological functions and their chemical behavior, are different. Chitosan has a nitrogen content of 6.80% or higher. Generally, when the content of N-acetyl groups is higher than 50%, chitin is considered, while for lower values, chitosan is considered [7].

By taking into account its molecular mass, chitosan can be split up in three different categories:

− low molecular weight chitosan, with a mass lower than 100 kDa;
− medium molecular weight chitosan, with a mass between 100 and 1000 kDa;
− high molecular weight chitosan, with a mass higher than 1000 kDa.

The molecular mass of chitosan affects its properties, so it is important to know it in order to make a strong correlation between chitosan type and any further application prior to chitosan modification [11,12].

The most important property of chitosan is its antibacterial activity. This feature reaches a new dimension in the context of the need to find new materials that have bactericidal effects on bacteria resistant to existing antibiotics. It is known that chitosan is a bactericide/bacteriostatic agent acting upon a various number of common bacteria, both Gram-positive and Gram-negative [5,13,14,15,16,17]. 

Chitosan has several advantages over other types of antimicrobial agents because it shows a higher antibacterial activity, a wider spectrum of antibacterial activity, and a lower toxicity towards mammalian cells [8,15,18]. The use of chitosan in medicine is very important. Over time it has acquired various uses, which are spectacular as areas of interest. In terms of the extremely diverse “bio” field, chitosan has been used in biotechnology [19,20,21,22,23], food preservation [13,24], drugs and pharmaceuticals [5,25,26], and gene therapy [27,28].

In theory, chitosan has a high biological activity with very wide applicability, but its poor aqueous solubility limits this theoretical advantage, including its antimicrobial behavior [8]. The antimicrobial action of chitosan is influenced by numerous intrinsic factors, such as its sources (crustaceans [29], insect shells [30], fungi [31]), concentration [32], the molecular weight that generates its type [33,34,35,36], polymerization degree [2,37], but also by external factors, including pH of the environment [33,38], the type and sensitivity of targeted microorganisms [14,16,33,39,40], the chemical composition of the substrate, etc. Many factors change the behavior of chitosan. The pathway to obtain chitosan derivatives is directly linked to their practical applicability, so different properties of these derivatives will determine their (re)activity.

**Figure 5:**
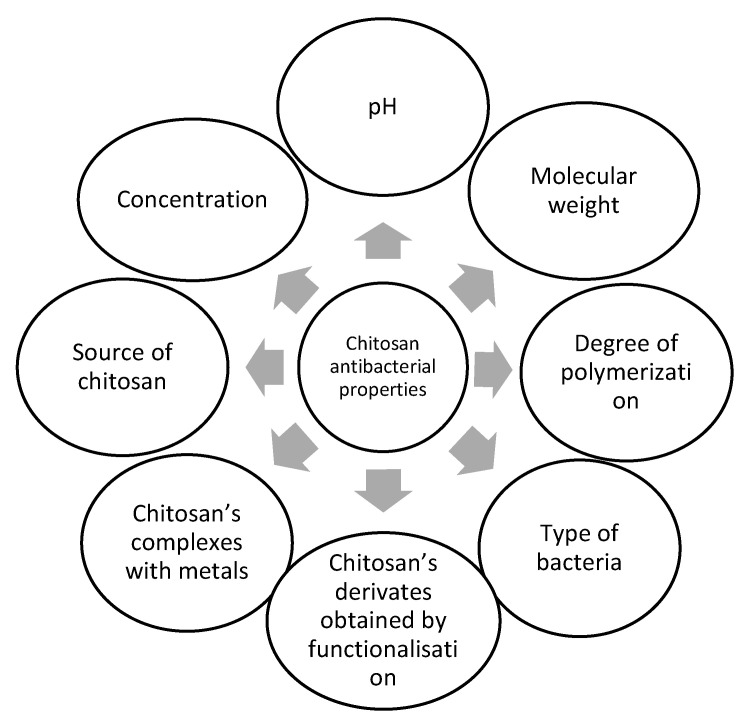
Intrinsic and external factors contributing to chitosan’s antibacterial activity.

Figure 5 shows the most important intrinsic and external factors influencing the antibacterial effects of chitosan.

It is difficult to rank these factors, taking in account their influence on the antibacterial activity of chitosan. We will discuss some of these factors further on. Therefore, as many factors as possible that may influence the antibacterial activity of chitosan must be considered.

### 1.1. Chitosan Sources and Their Contribution to the Antibacterial Activity of Chitosan

Chitin is the most abundant polysaccharide on earth, after cellulose; it is the main structural polymer found in the fungal cell wall [1]. It is also present in the exoskeletons of arthropods [41] and insects [30]. Chitosan, the main derivative of chitin, can therefore come from fungi and the exoskeleton of crustaceans or insects.

Some studies have mentioned that growing certain mushrooms could provide an effective source of chitosan for industrial applications [30]. Many researchers have concluded that chitosan can be extracted not only from *Zygomycete* fungi, but also from non-*Zygomycete* fungi [31].

Chien et al. reported that crude chitin from crab shells did not show any antimicrobial activity, but chitin from mushroom exhibited an inhibitory effect on bacterial growth, compared with chitin from crab shells [42].

Their results showed that the antimicrobial activity of fungal chitosan was lower than that of chitosan obtained from crustacean shells. However, fungal chitosan, similar to crustacean chitosan, exhibited better inhibitory effects against Gram-positive bacteria compared with Gram-negative bacteria [33].

It has been demonstrated by Byun et al. that chitosan prepared from the entire crab shell and the shell of the crab leg shows major differences in terms of physical–chemical and functional characteristics. For example, chitosan prepared from crab shell had a significantly higher nitrogen content, degree of deacetylation, solubility, and viscosity and improved antibacterial activity than chitosan prepared from crab legs [29].

On the other hand, chitosan oligomers and polymers from different sources present different antibacterial activities. They have been tested against food borne pathogens, and the results demonstrate that the source, degree of deacetylation, and molecular size of chitosan must be selectively chosen to control food borne target pathogens [33].

### 1.2. Influence of Chitosan Concentration on the Antibacterial Effect

A lot of experiments suggested that chitosan can inhibit bacterial growth at different concentrations [43]. Usually, the required concentration of chitosan to inhibit bacterial growth is correlated with the acetylation degree of chitosan; a solution exhibiting 7.5% acetylation degree was more effective than that exhibiting 15% acetylation degree [43,44]. 

Only at lower concentrations does chitosan bind to the negatively charged cell surface, especially of Gram-negative bacteria. In this case, it interferes with the cell membrane permeability; thus, intracellular components will be externalized, leading to cellular death. On the other hand, at higher concentrations, chitosan that is positively charged due to amino groups may coat the cellular surface, and the intracellular components are blocked in the cell. In addition, in the case of Gram-positive bacteria, the positively charged bacterial cells and the positively charged chitosan will have a repelling effect on each other, preventing agglutination [33,45].

Liu et al. evaluated the effects of the molecular weight and concentration of chitosan against *E. coli*. Different molecular weight chitosans (5.5 × 10^4^ to 15.5 × 10^4^ Da) in various concentrations (20 to 1000 ppm) were used. All chitosan samples with molecular weight from 5.5 × 10^4^ to 15.5 × 10^4^ Da had good antimicrobial activities at high concentrations (>200 ppm), and all samples at low concentration (20 ppm) could promote the growth of *E. coli* [32]. This might be explained by the fact that bactericidal effects of high concentrations of chitosan can occur through the flocculation of bacteria. Low concentrations of chitosan did not exert this effect, promoting instead the survival of bacteria by favoring their reproduction [32,46].

### 1.3. Environment pH Influence on the Antibacterial Effect of Chitosan

Environmental pH is one of the most important factors affecting the antimicrobial activity of chitosan and its derivatives.

pH primarily affects the solubility of chitosan, but it also affects the electrical charges of the chitosan molecule. This property enables chitosan molecules to bind via electrical interactions [47].

**Figure 6:**
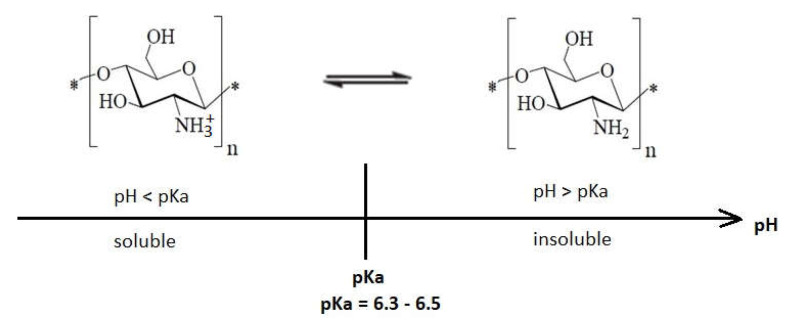
The pH influence upon chitosan solubility according to [57].

It is well known that native chitosan is soluble in organic acids at pH lower than 6, but it is insoluble in water, organic solvents, and alkaline medium (Figure 6). Preparation of different water-soluble chitosan salts is possible by its neutralization with hydrochloric acid, acetic acid, lactic acid, or formic acid [43]. Its solubility in diluted aqueous solutions can be correlated with the conversion of glucosamine units into the soluble form of R-NH_3_^+^. Experimental data proved that water insoluble chitosan shows antimicrobial activity in acidic medium, being appropriate for use as a preservative in acidic foods [48].

At pH lower than pKa, chitosan molecules are protonated due to the high density of amino groups (–NH_3_^+^) that get converted to the quaternary form, giving a positive charge to the polymer, which increases the intermolecular electric repulsion, resulting in a polycationic macromolecule [38]. Chitosan has polycationic behavior at pH < 6, which makes it soluble in water. It was observed that while pH decreases, the adsorption of chitosan on bacterial surfaces will increase. These are the requirements to interact with negatively charged substances like proteins, fatty acids, and phospholipids, which are components of the bacterial cell [33,38,49]. Thus, the interaction between positively charged chitosan molecules with negatively charged residues on the bacterial surface is possible, and in this way, the cell permeability is perturbed, which ultimately leads to bacterial death [16,33,40,50]. In all mechanisms that explain the antibacterial effects of chitosan molecules, the cationic charge is considered to be responsible for efficient binding of chitosan to the anionic components that are present at the level of bacterial membranes [45,48,51,52,53,54,55,56]. The interaction between protonated chitosan and negatively charged cell membranes is the most common mechanism that explains cellular death, referred as the “antibacterial effect” of chitosan.

There is the problem of the pH difference between the physiological pH of most bacterial cells (which is a neutral pH) and the pH at which chitosan is soluble. The physiological pH of the cell being neutral, chitosan molecules precipitate, and chitosan molecules remain on the surface of the bacterial cell, acting like a layer that blocks the ion exchange channels from the cellular wall, which are essential for the good survival of the microbial cell. This mechanism destabilizes the cell wall morphology and functions, causing severe damage to cell constituents and, in the end, the cell will die [48].

This mechanism of action is not unique; the antibacterial effect of chitosan is the result of a string of molecular processes that cause multiple damages, leading to cellular death. 

Most research has shown that when the pH of the medium is below pKa, the polymer has an increased antimicrobial effect [35,43]. It seems that the presence of positive charge on the structure of the polymer, rather than its solubility depending on the pH range, is the critical factor for expressing antimicrobial activity [58].

A series of studies has shown that chitosan derivatives are active towards Gram-positive and Gram-negative bacteria only when the degree of substitution is low; chitosan derivates should have a higher number of protonated amino groups [14,39,59,60,61,62,63].

Not only the presence of positive charge is enough, but a decisive role in exerting the bactericidal effect of chitosan derivatives is played by the location of the cationic charge, compared to the polymer backbone structure. In the case of a series of compounds with different spacing lengths of substituted carbon, the inhibitory effect of the compounds decreased if the distance between the backbone of the polymer and the cationic position increased. Most researchers conclude that the antimicrobial effect seems to be highest if cationic fragments are closer to the polymer backbone, and it tends to decrease when the functional groups are present at an increasing distance from the polymer chain. This effect is clearly observed when the same functional group is bounded to the polymer by chains of different lengths [8,62,64].

At pH higher than pKa, chitosan tends to lose its positive charge, and it precipitates due to deprotonation of amino groups, becoming insoluble. This is explained by the fact that the majority of amino groups become uncharged at pH close to 7 [38,58]. Although at pH > pKa chitosan is deprotonated, it still remains reactive, having the possibility to form gels or protective films [56,65,66].

Another study concluded that at pH = 6.2, a stronger biocidal effect was observed, which is related to the particular pKa of chitosan (pKa = 6.3–6.5) [67], obviously very close to a pH of 6.2. At this pH, the amount of positively charged amino groups is about 75% in chitosan, while at pH 7.4, this quantity represents approximately 10% [66].

### 1.4. The Molecular Weight Contribution to the Antibacterial Effect of Chitosan

Based on molecular weight, there are three types of chitosan:

✓ low molecular weight (LMw) chitosan, named also “oligo-chitosan” or “short chain chitosan” (molecular weight < 50 kDa);
✓ medium molecular weight (MMw) chitosan, with molecular weight between 50 kDa and 250 kDa;
✓ high molecular weight (HMw) chitosan, with molecular weight > 250 kDa [6,35,38].

Almost all studies reported a correlation between the bactericidal effect of chitosan and its molecular weight [68,69]. Oligosaccharides and D-glucosamine have no antibacterial activity. In some studies it is suggested that a minimum molecular weight of 10 kDa is required for a bactericidal effect [70].

The relation between the bactericidal effect and the molecular weight is influenced by the type of implied bacteria [36]. HMw chitosan cannot cross microbial membranes; thus, it remains on the cellular surface, blocking nutrients to be transported inside microbial cells, leading to cell lysis. A dissociated solution of HMw chitosan molecules can bind to the cell membrane, modifying its permeability. Dissociated solutions of LMw chitosan molecules can bind to DNA while penetrating the cell nucleus, inhibiting the synthesis of mRNA [14,35,68,69].

In various studies on several bacteria, such as *Staphylococcus aureus, Bacillus cereus, Klebsiella pneumoniae,* and *E. coli*, it was found that the lower the molecular weight (LMW) of chitosan, the higher the antibacterial effect [14]. This is attributed to the size and conformation of chitosan particles that appear to play an essential role in understanding the effectiveness of low molecular weight chitosan. The mobility, attraction, and ionic interaction of small chains are easier than those of large ones. The priority is to facilitate the adoption of large conformations, which can be efficiently bound on the membrane surface [34,71].

### 1.5. The Contribution of the Degree of Polymerization to the Antibacterial Effect of Chitosan

The degree of polymerization (DP) and the degree of deacetylation (DD) represent two intrinsic parameters that influence directly the chitosan chain structure. These are basic physico-chemical parameters that determine the use of chitosan in different applications.

The degree of acetylation can be written in the following way:

$\%DA=\frac{8.695-\%N}{8.695-6.896}*100$ where, according to [7],

8.695 = the percentage of nitrogen in fully deacetylated chitosan,

6.896 = the percentage of nitrogen in fully acetylated chitin,

and

%DA=100−%DD

Thus,

%DD=100−%DA

Chitosan, being actually a mixture of homologue units of N-acetyl-d-glucosamine monomer units and d-glucosamine monomer units, at a different degree of polymerization, is not a pure polymer. Chitosan is relatively insoluble in water until it is broken down into oligomers with ≤7 sugar residues or seven degrees of polymerization [37].

In this context, by polymerization, it is gradually possible to increase the molecular weight of chitosan, and this influences the viscosity and solubility of chitosan [35,36,39]. The solubility in water increases with the decrease in the DP and with the increase in DD. In acidic medium, the amino groups have a positive charge, and this causes the deacetylated chitosan to easily dissolve in aqueous acidic medium [2,48].

Because chitosan can be obtained from chitin, studies have also focused on the degree of polymerization of chitin. A degree of polymerization of less than four for chitin does not seem to have an important biological role, while chitins with DP > 6 seem to be much more active [3,72,73].

Graft copolymerization onto chitin and chitosan is possible. Using this procedure, it is possible to generate various designs of the molecule, generating new types of materials between natural polysaccharides and synthetic polymers [5]. The properties of these new materials can be controlled by molecular structure or length and number of attached side chains. For example, crosslinking affects the permeability characteristics [51,74], and through covalent or ionic interactions results in the formation of chitosan hydrogels that are used in drug delivery systems [74,75].

It was demonstrated also that an increased antimicrobial activity needs a certain number of monomeric units, and this value depends on other factors, such as the nature of the substituents that are attached and the types of microorganisms that are tested [62].

Chitosan oligomers have excellent solubility. In addition, oligomeric chitosans (pentamers and heptamers) have better antifungal activity than larger units [43]. Longer polymers of chitosan are water soluble in solutions at pH 7 or lower, and they are also soluble in dilute organic acids such as lactic and acetic acids. The solubility can be maintained by slowly adjusting the solution back to around pH 7 with NaOH [76].

### 1.6. The Contribution of the Type of Bacteria to the Antibacterial Effect of Chitosan

The first step in understanding the antibacterial activity of chitosan on different types of bacteria was made by Allan and Hadwinger in 1979 [77]. Over time, the entire antibacterial activity of chitosan itself was attributed to the amino groups that are directly influenced by DP degree and DD degree.

Due to the different composition of the Gram-positive and Gram-negative cell walls, the interaction of chitosan is different with these two types of bacteria. In some studies, researchers found that the bactericidal effect was more important on Gram-negative bacteria than on Gram-positive ones [40] because of the higher affinity of amino groups for anionic radicals present in the cell wall. In other studies, Gram-positive bacteria were considered to be more sensitive to the antimicrobial activity of chitosan, as a consequence of the Gram-negative outer membrane barrier [16,78]. These discrepancies should not surprise us, given that the bactericidal activity of chitosan is influenced by so many factors, both intrinsic and extrinsic.

Antimicrobial activity of chitosan is considered to occur when the compounds are absorbed onto the **surface of the bacterial cells**. This is followed by an increase in the permeability of the lipid cell membrane, and essential compounds leave the cell, leading to cellular death [33].

Although several mechanisms for the antibacterial activity of chitosan and chitosan derivatives have been suggested, the exact mode of action is still not known in detail. However, there is clear evidence that these compounds express molecular-level interactions with the cell membrane [14,61,79]. Usually, ionic and/or hydrophobic interactions are considered to be responsible for the damage or breakage of cell membranes [69,80]. Based on these considerations, the hydrophilicity of Gram-negative bacteria is significantly higher than that of Gram-positive bacteria, making them more sensitive to the action of chitosan. Thus, following the action of an antibacterial agent, the cell wall of Gram-negative bacteria passes several morphological changes compared to Gram-positive ones. Determinant for this is the density of electrical charges on the surface of the bacterial cell, which is actually crucial for the amount of adsorbed chitosan. The more chitosan is adsorbed, the more obvious are the changes in the structure and permeability of the cell membrane [81].

Electronic microscopy proved that chitosan induces extensive cell surface alteration while covering the bacterial outer membrane with vesicular structures, causing alterations of the barrier function of the cell membrane [69,82,83].

Chitosan possesses a polycationic character and interacts predominantly with the anionic wall components of bacteria (especially lipopolysaccharides and proteins). The consequences of this interaction are represented by externalization of intracellular components and the impossibility for nutrients to enter into the bacterial cell, nutrients being bound to metals [84].

Amino groups, via chelating mechanisms, can react with the metal ions. Because amino groups are protonated in an acidic medium, they can cause electrostatic attraction of anionic compounds, such as anionic residues or proteins, by altering their normal functioning; but, simultaneously, the affinity of the absorbent to bind metal cations is reduced [85].

**Figure 7:**
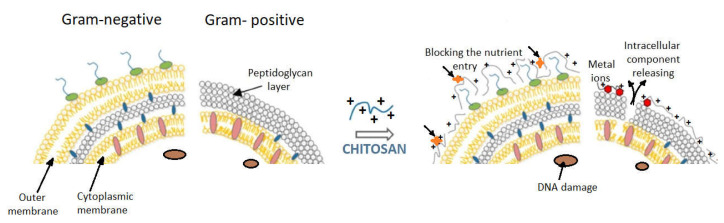
Models proposed for chitosan action upon Gram-positive and Gram-negative bacteria (from Kravanja et al., 2019 [55]).

The membrane of **Gram-negative bacteria** is a thin two-dimensional structure containing a peptidoglycan layer that forms a hydrophilic surface (Figure 7). The cytoplasmic membrane is made of lipopolysaccharides, lipoproteins, and phospholipids. When the protonated amino groups of chitosan meet a certain anionic bacterial surface (carboxylic residues, phosphate residues, etc.), electrostatic binding is possible, especially if chitosan is of a low molecular weight type. Subsequently, cell permeability is affected, and osmotic stability of the bacterial wall is decreased. The complexes resulting from this interaction affect the barrier properties of the cytoplasmic membrane; they enter into the cell and interfere with the physiological bacterial processes or cause a leakage of enzymes and nucleotides from bacteria [16]. In conclusion, in Gram-negative bacteria, the external membrane layer works as a barrier against hydrophobic residues and macromolecules, explaining the resistance of the Gram-negative bacteria against hydrophobic antibiotics.

Chitosan exerts a chelating effect [85], binding essential metals and thereby inhibiting microbial growth. It is well known that chitosan has excellent metal-binding abilities, using charged amino groups that interact with metals [85,86,87]. This type of interaction between amino functions and divalent ions within the microorganism cell wall (Ca^2+^ or Mg^2+^) prevents the production of toxins and inhibits bacterial growth [69,88]. 

Bacterial membrane permeability is importantly influenced by the molecular size of chitosan and the pH of the medium. These factors were described above.

The cellular wall of **Gram-positive bacteria** is a three-dimensional layer, consisting especially of peptidoglycans. Gram-positive bacteria have no outer membrane (Figure 7). 

One proposed mechanism for the bactericidal effect of chitosan on Gram-positive bacteria is its direct blocking capacity, preventing nutrients and oxygen from entering the intracellular space [33]. This mechanism is suitable for higher molecular weight chitosan, which forms a polymer membrane on the surface of the bacterial cell.

Another bactericidal mechanism of chitosan with low molecular weight derives from the interaction with DNA that inhibits mRNA and protein synthesis after entering the nuclei of microorganisms. This was demonstrated in the case of *E. coli*, where intracellular chito-oligomers were observed, and these probably prevent DNA transcription [89].

In conclusion, the different sensitivities of Gram-positive bacteria compared with Gram-negative ones, as a result of chitosan action, is primarily due to the difference between the cell wall structures of the two categories of bacteria. Gram-negative bacteria have three barrier membranes (the hydrophobic outer membrane, the peptidoglycan layer, and the cell membrane), while Gram-positive bacteria have only a thick peptidoglycan layer (that contain teichoic acid and is negatively charged). In addition to these structural differences, intrinsic (molecular mass, degree of deacetylation) and extrinsic factors (concentration, pH, contact time) always occur in the manifestation and magnitude of the bactericidal effect of chitosan or its derivatives on different types of bacteria.

### 1.7. The Chitosan Derivatives’ Contributions to the Antibacterial Effect 

Chitosan solubility in water is limited. It has been synthetically modified, resulting in improved aqueous solubility and increased antibacterial activity. Based on the type of functional groups that are attached to the polymer backbone, many antibacterial chitosan derivatives exist.

In most cases, functionality of chitosan is provided by chemical reactions that require certain conditions to be accomplished. However, there are some situations where it is sufficient to create reaction conditions that do not actually involve a chemical reaction between components. This is the case for chitosan functionalized by impregnation with various pendant groups; it is necessary to put chitosan in contact with the extractant for a certain time, after which the material is filtered, washed, and dried.

In further sections, we will describe the most important methods of chemical functionalization of chitosan, referring especially to the hanging groups that can be attached to the chitosan chain. Our objective is a better understanding of the mechanisms that can explain the bactericidal effects of chitosan functionalized by impregnation.

The antibacterial effects of chitosan and its derivatives have been reported by many authors [10,14,90,91]. It was demonstrated that both native chitosan and its derivates have bactericidal effects, but there are evident differences among them.

**Figure 8:**
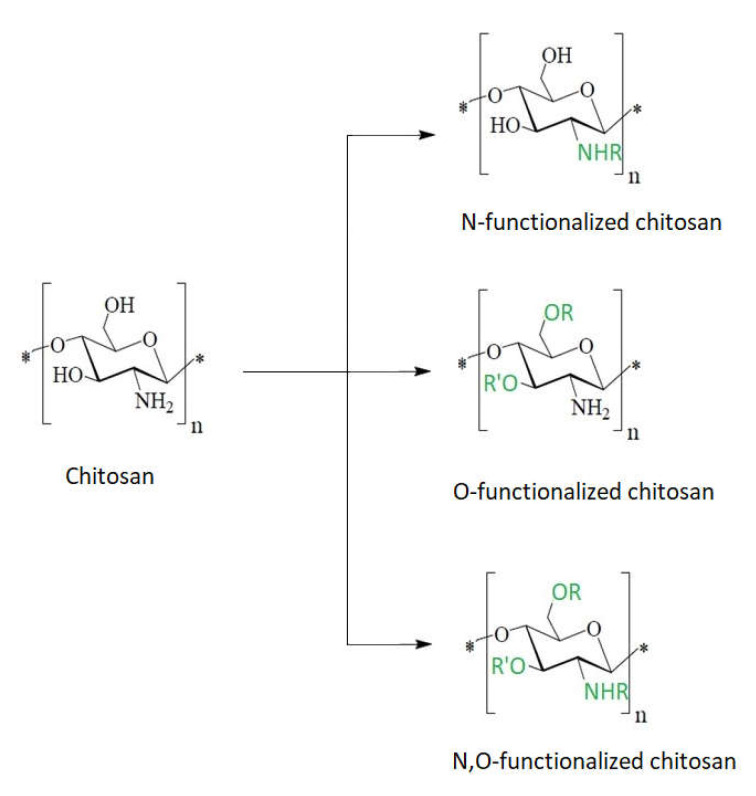
Schematic chitosan functionalization.

The presence of –NH_2_ and –OH nucleophilic functional groups allow chitosan to be modified either at the amino group or at the hydroxyl group (Figure 8). In this way, chitosan becomes a support material for the synthesis of another material (chitosan derivative) that will exhibit superior properties.

Chitosan derivatives with substituted functional groups for both the –OH and –NH_2_ reactive center have an increased bactericidal effect against both Gram-positive and Gram-negative bacteria compared to chitosan functionalized only at a single reactive center [83].

Due to the poor solubility of chitosan in aqueous medium, synthetic modifications of chitosan have mostly been carried out in acidic–aqueous media or under heterogeneous conditions where the polymer is only partially dissolved in the reaction medium. These conventional methods usually allow one to obtain products that can be substituted at all three reactive centers of chitosan (the 2-amino group and the 3- and 6-hydroxyl groups), and this ultimately results in a heterogeneous product or a product having a low degree of substitution. To overcome these issues, different types of groups were introduced to protect either the amino group or the hydroxyl groups [62]. In this case the synthesis of chemically modified chitosan derivatives can be done selectively by multiple step reactions.

**To protect the amino group,** the most used protecting group is phtaloyl [92,93].

**To protect the hydroxyl groups,** the most useful protecting groups are acetyl, triphenylmethyl, and trimethylsilyl groups [93,94,95].

### 1.8. Chitosan Containing Alkyl Groups

It was observed that the bactericidal activity is proportional to the chain length of the **alkyl substituent** due to the contribution of the hydrophobic properties of the derivates. The hydrogen bond between molecules was significantly weakened when chitosan was modified by alkyl groups. These results prove that hydrophobicity and cationic charge of the substituent affect directly the antibacterial activity of the derivative [80]. If an alkyl chain is introduced within a modified chitosan (like N-methylene phosphonic chitosan), the new derivative has both hydrophobic and hydrophilic chains in its structure. This is why the presence of an alkyl chain in the structure of a chitosan derivative is considered responsible for the weakening of hydrogen bonds, which ensures good solubility in organic solvents, even if chitosan itself is not soluble in water under normal conditions [96].

Generally, alkyl groups can be introduced at –OH or –NH_2_ positions of chitosan, in the presence of alkyl halides (i.e., ethyl, butyl, dodecyl halides) and a strong base.

Usually, if chitosan is modified with halogenated alkanes of different chain lengths, the alkylation process occurs at the –NH_2_ of the C2 position.

**Figure 9:**
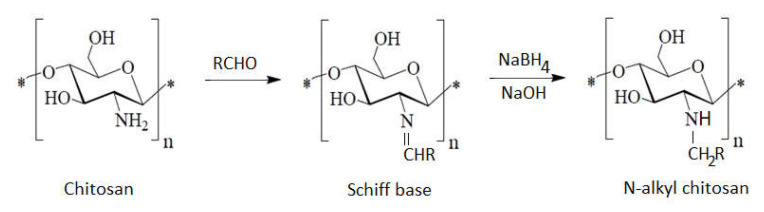
N-alkyl chitosan obtained via Schiff base, according to [97,99].

If selective alkylation at the –NH_2_ position of chitosan is needed in order to obtain N-alkyl chitosan, initially a reductive amination (formed Schiff base) takes place, and then a reduction to the final product occurs (Figure 9). This method is applicable in several reactions between substituted aldehydes (benzaldehydes or ketones) with chitosan [5,78,97,98].

This method is also used to protect the amino groups within chitosan (via Schiff’s base production) and to allow hydroxyl groups to be modified [8,100]. To obtain O-alkylated chitosan derivatives, firstly the protection of the amino group is needed (via Schiff’s base), and then the removal of the protective group to obtain the alkylation product occurs. 

The alkyl substituent can also be found in different forms (hydroxyalkyl, carboxyalkyl), each of them with specific properties.

In the case of **hydroxyalkyl substituents**, the reaction takes place at the amino or hydroxyl groups, resulting in N-hydroxyalkyl or O-hydroxyalkyl chitosan. The ratio of the N/O substituent is generated by the choice of the catalyst (NaOH or HCl) and by the reaction temperature. Thus, to achieve N-hydroxypropylation, no catalyst is needed. If the reaction takes place in the presence of acid catalysis, mainly N- and O-alkylation products are obtained. Basic catalysis will lead to O-alkylation products being obtained [99].

Hydroxyalkyl derivatives of chitosan are generally obtained by treating chitosan with epoxides, under heating. In this type of reaction, pH is a critical factor; high pH determines hydrolysis of the reagent. This is the reason to use slightly alkaline conditions and low NAOH concentrations for the synthesis [62].

**Figure 10:**
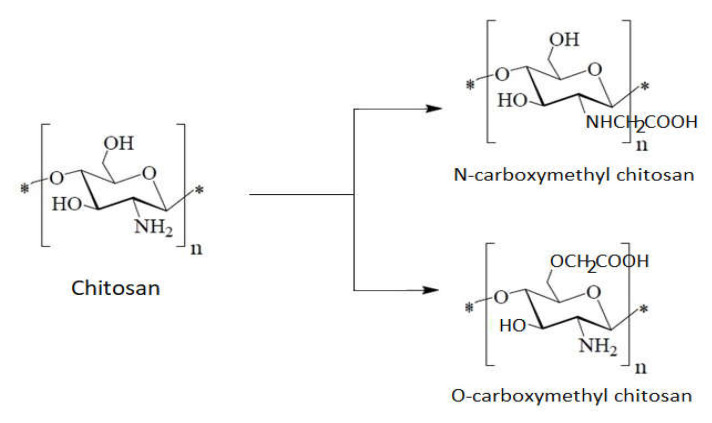
Schematic carboxyalkylation of chitosan.

In the case of **carboxyalkyl chitosan** (Figure 10), acidic groups are introduced at the amino groups of chitosan, and an amphoteric electrolyte mixture is obtained that contains cationic and anionic charges. To obtain a variable charge density on the molecular chain that generates a pH dependent behavior, it is necessary to vary the degree of carboxyl group substitution [99].

Using different reaction conditions with monohalocarboxylic acid, it is possible to obtain both N- and O-carboxyalkyl [101]. To obtain selectively only N-carboxyalkyl derivates, carboxaldehydes must be used via a reductive amination route [99].

**Figure 11:**
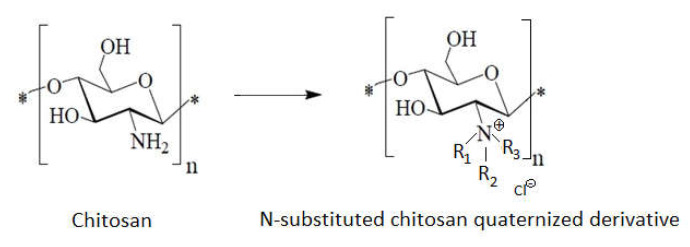
General structure of N-alkyl chitosan derivatives.

Alkyl chitosan can be used for **quaternization** (methylation) (Figure 11). Several experiments shown that quaternized chito-oligomers exert antibacterial activity [33,81,95].

Different degrees of quaternization of amino groups in chitosan can be obtained via methyl iodide of N-methyl pyrrolidinone, in alkaline medium [99].

Quaternized derivates of chitosan, such as N,N,N-trimethyl chitosan chloride, are more soluble in water than chitosan in a wide pH and concentration range. These demonstrate that chitosan acts as an absorption enhancer for test drugs [61].

On the other hand, quaternized derivates of chitosan (i.e., N,N,N-trimethyl chitosan chloride) are more suitable for the collection and delivery of negatively charged DNA than plain chitosan because of a stronger basic property of the quaternary ammonium group. This is the reason to affirm that the most important application of alkylated chitosan resides is DNA delivery. The transfection efficiency of alkylated chitosan is higher because it enters more easily into the cell, facilitated by hydrophobic interaction and by an easier unpacking of DNA due to the weakening of electrostatic attractions between DNA and alkylated chitosan [101]. Quaternary chitosan salt determines the unpacking of the chitosan molecule, making the molecule slightly flat, which allows for its entrance into the bacterial cell more easily.

Introducing several functional groups, like trimethyl, quaternary alkyl groups will give to the polymer a permanent positive charge that improves chitosan’s solubility in aqueous medium and allows the measurement of its bioactivity at pH 7 [29,39,61]. Alkyl chitosan derivatives have good adsorption and chelating properties towards various metal ions. Long-chain derivatives of N-alkylated chitosan have amphiphilic properties, which play a very important role in the adhesion of these compounds to the bacterial cell membrane [4]. 

However, the most important application of alkylated chitosan is in the delivery of DNA. This has been demonstrated by the use of dodecyl chitosan in experiments [89]. It has been proposed that the higher efficiency of alkylated chitosan in genetic transcription is attributed to its increased entry into cells. Transcription is facilitated by hydrophobic interactions of alkylated chitosan with an easier breakdown of DNA from alkylated chitosan due to decreased electrostatic attractions between DNA and alkylated chitosan. As the alkyl side chain increased in alkylated chitosan, the efficiency of transcription increased and subsequently became relatively constant after the number of carbon atoms in the side chain exceeded eight [101].

### 1.9. Chitosan Containing Aromatic Groups

Chitosan is a multinucleophilic polymer because of the presence of –NH_2_ and –OH groups It can be also acylated at both groups forming either an amide or an ester. An acylation reaction can introduce **aromatic groups** to the chitosan skeleton to the amino group.

**Figure 12:**
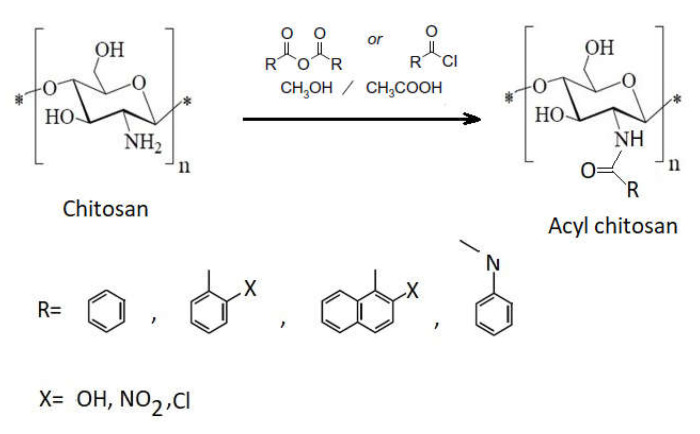
Schematic acylation of chitosan.

First, acylation takes place at the amino group (Figure 12), because it is more nucleophilic than the hydroxyl group. The condensation reaction occurs between the primary amine and the carbonyl group, leading to the formation of a Schiff base (–RC=N–), an N-acylated derivative of chitosan, showing potential antimicrobial and biodegradable properties [100] due to the hydrophobic characteristics of the added alkyl chain.

By N-acylation of chitosan with a longer fatty acid chain (C6–C16), the hydrophobic character of the derived product increases, and important changes take place in its structural properties. Unmodified chitosan has a low degree of molecule ordering and a low degree of molecule compression. If chitosan is acylated with a short chain fatty acid (<C6), the chitosan derivative has similar properties, but some excrescences have been observed in its structure. If the acylation takes place with longer chain fatty acids (C8 or higher), the molecule has a more orderly structure and the excrescences observed in the case of chitosan acylation where short chain fatty acids have disappeared [96,102].

O-acyl chitosan can be obtained if reaction conditions are modified and a protection of amino groups is achieved [4]. In this case, two main benefits can be described: the organic solubility (due to hydrophobic group), and the presence of ester linkage that further on can be hydrolyzed by enzymes. Thus, most O-acyl chitosan derivates are biodegradable under the action of different enzymes (lipase, glycosidases, etc.).

Antimicrobial activities of chitosan and of the Schiff bases of chitosan were investigated against *S. aureus*, *B. subtilis*, *E. coli*, and *A. niger*. It was observed that Schiff bases of chitosan have better antimicrobial activity than chitosan [78,100].

Chitosan Schiff base derivatives contribute to the increase of the antimicrobial activity of chitosan, because the carbonyl groups in the aldehydes or ketones with which chitosan reacts can efficiently couple with –NH_2_ groups of chitosan, forming the corresponding Schiff base with the characteristic imine group (–RC=N–). This leads directly to changes in the molecular structure of chitosan, improving its hydrophilicity, as well as to charging the molecule with positive ions, which results in better antibacterial activity compared to unmodified chitosan [78,98].

The spatial hindrance and hydrophobic forces in the aromatic substituent groups of chitosan derivatives have important roles in the crystallinity and thermal stability of the final products [103]. Aromatic chitosan derivatives, such as chitosan tripolyphosphate nanoparticles, exhibit some attractive properties, such as delivery of macromolecules in muscles, having pharmaceutical and medical applications [104].

In recent research, the mechanical properties of chitosan were attempted to be improved. The results showed that for the strong binding of chitosan to the surface of cell membranes, which automatically involves the improvement of its mechanical properties, the presence of functional groups such as amino and phenolic hydroxyl ones is sufficient. The same studies showed that the presence of these groups onto chitosan determines an improved tensile strength [102,104].

### 1.10. Chitosan Derivative with Sulfur as Heteroatom

The characteristics of chitosan, such as the fact that it is a cationic and hemostatic polymer, which is insoluble in water at a high pH value, can be modified by sulfating their amino groups, making the macromolecule anionic and soluble in water; in addition, the obtained molecule acquires anticoagulant properties, showing structural similarities with heparin [57].

The sulfonate group can be attached directly to the amino group of chitosan, resulting in products such as chitosan sulfate (–NH-SO_3_^−^) [105]. It can also be introduced using compounds containing sulfonate groups (R-SO_3_^−^), resulting in sulfonated derivatives (–NH-R-SO_3_^−^) [106,107].

When chitosan reacts with sulfating agents, the major substitution is at the C6-OH group. In this case, the mechanism for obtaining chitosan derivatives with sulfur is an electrophilic substitution reaction in which the proton is substituted with the –SO_3_H group [97]. 

Chitosan sulfonated derivatives are soluble in water and have an anionic behavior in nature; they exert anticoagulant properties and also antiviral and antibacterial properties [108,109].

**Figure 13:**
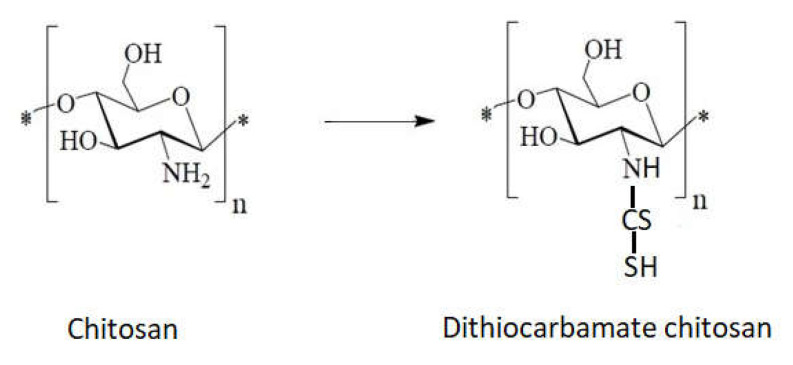
Schematic reaction of chitosan with carbon sulfur.

The sulfur heteroatom can be introduced into chitosan backbone by many methods:

1. Direct reaction of chitosan with carbon sulfur (Figure 13).

Chemically modified chitosan, by incorporating functional groups of dithiocarbamate, is selective for various metal ions (Ag, Au, Pd) with maximum adsorption capacity for Ag. This type of chitosan derivative has a higher adsorption capacity than chitosan derivatives obtained by functionalizing chitosan with chelating resins containing the same functional groups [110].

**Figure 14:**
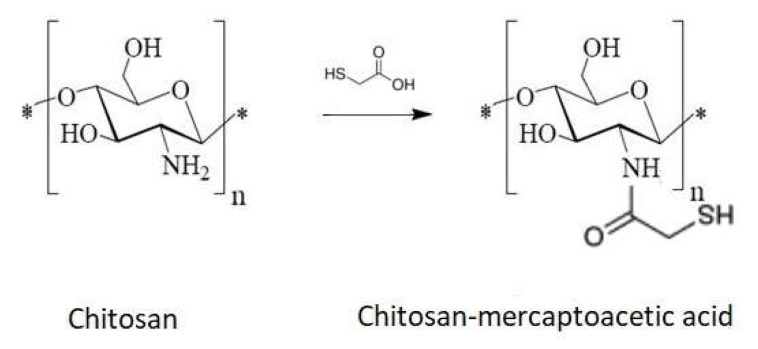
Schematic of chitosan sulfonation via mercaptoacetic acid.

**Figure 15:**
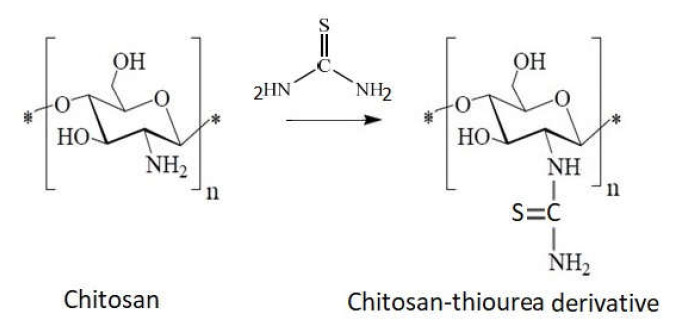
Schematic of chitosan sulfonation via thiourea.

**Figure 16:**
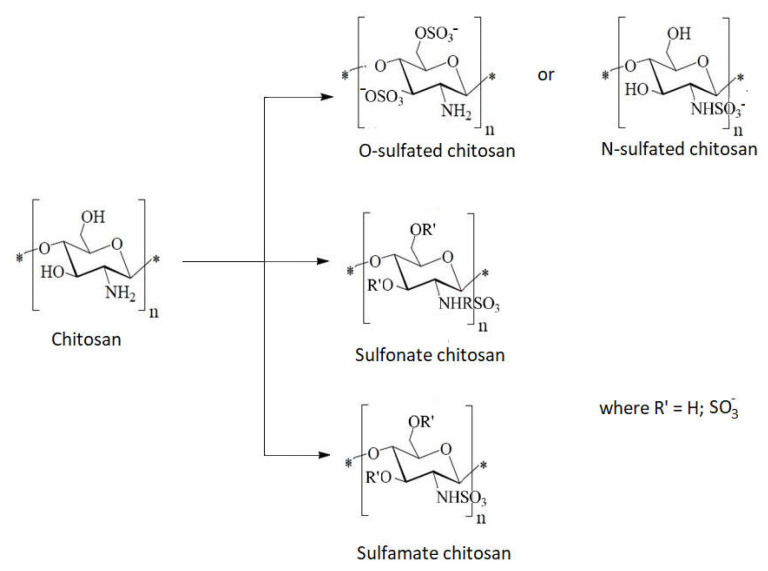
Schematic types of sulfonated chitosan derivatives.

2. Direct reaction with mercaptoacetic acid (Figure 14)

3. Grafting of thiourea (Figure 15) via a cross-linking agent or by a chitosan reaction with ammonium thiocyanate [111,112].

4. Grafting of sulfonic groups onto chitosan (Figure 16)

**Figure 17:**
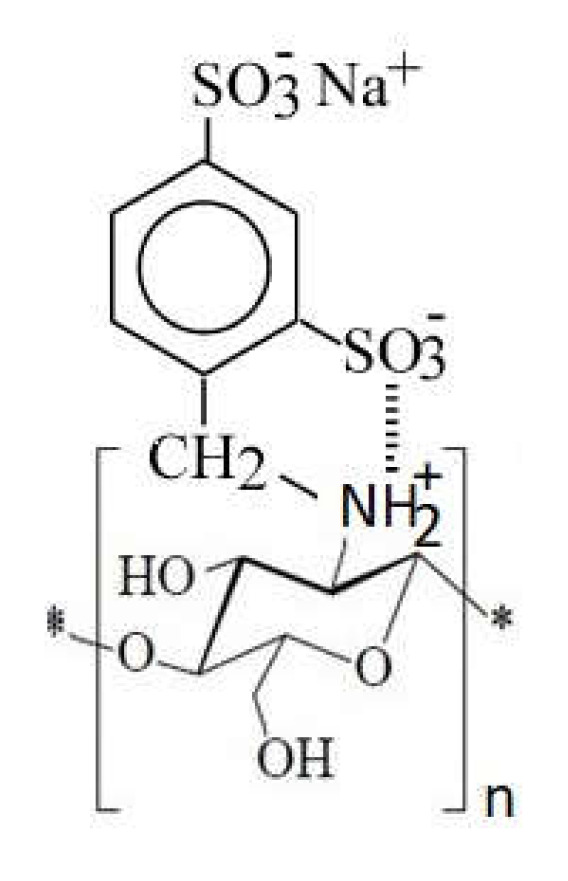
Schematic intramolecular interaction in chitosan sulfonated derivatives.

Due to the amphoteric character, which causes intramolecular interactions between the amino and the sulfonate group, monosulfonated derivatives of chitosan are not suitable for adsorption (Figure 17) [110].

It is therefore necessary to protect the chitosan amino groups (i.e., with benzyloxycarbonyl), to increase its adsorption capacity by reducing intramolecular interactions. Protected monosulfonated derivatives of chitosan have higher adsorption capacities than disulfonated derivatives [110].

Anticoagulant properties of sulfated chitosan derivatives are attributed to the interaction between the negative sulfate groups and the positive chain of chitosan. Sulfate functionalization of chitosan leads to a structure resembling that of heparin. These chitosan derivatives may be used in biomedicine [25,113].

Recently, it has been demonstrated that the incorporation of sulfate groups into the structure of chitosan improves its compatibility with blood and increases its anticoagulation effects by its complexing ability with blood [97,106,114]. Sulfur chitosan derivatives have increased anticoagulant activity. Quaternized chitosan derivatives have increased solubility in water, but a low anticoagulant effect. In these situations, in order to obtain a sulfur chitosan derivative having good anticoagulant properties, an N-alkyl chitosan derivative is preferred, and not a quaternary salt of chitosan sulfonate [97].

### 1.11. Chitosan Derivative with Phosphorus as Heteroatom

To improve the water solubility of chitosan and to maintain its molecular weight, chitosan can be modified by phosphorylation. At pH > 6.5, phosphorylated chitosan has deprotonated phosphate groups that increase the solubility in water, which is necessary for various biological and industrial applications. 

As a practical applicability, chitosan phosphate derivatives are used to highlight their bactericidal and osteoproductive properties [115,116]. Chitosan derivatives that contain phosphate groups are amphoteric. This is the result of the interaction between the negative charge of the phosphate group and the positive charge of the amino group. In this way, by chitosan’s phosphorylation, a series of derivatives with orthopedic applications are obtained, due to the cation exchange properties of phosphate groups. Phosphate groups bind calcium ions, which can induce the formation of a layer of calcium phosphate [116]. This technique is the one used for dental implants.

Another application of chitosan derivatives with phosphorus is in tissue regeneration. Phosphate groups, due to their ion exchange properties, are sites of specific binding of biologically active species (i.e., cell growth factors that subsequently cause tissue regeneration) [116]. Thus, amphoteric chitosan-based surfaces are obtained that can be used as a combinatorial strategy to promote cell attachment and proliferation, while immobilizing signaling molecules.

**Figure 18:**
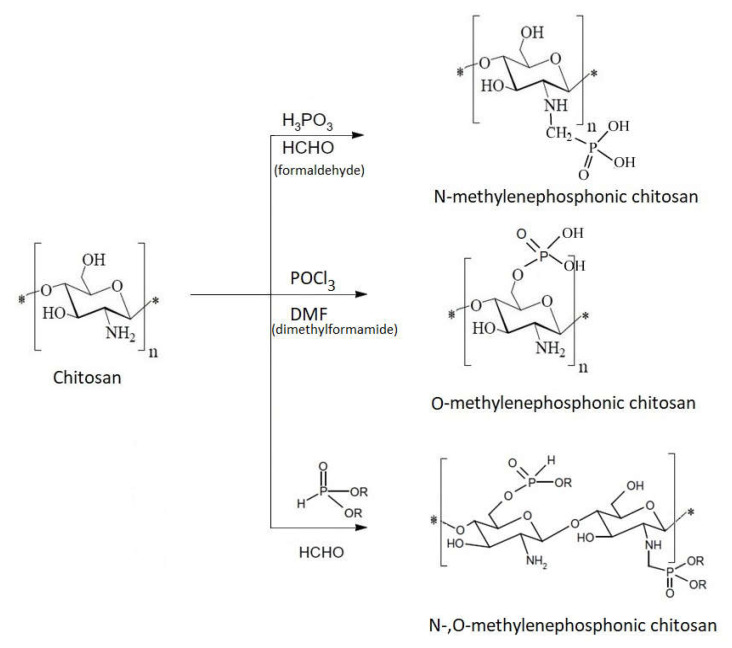
Schematic phosphorylated chitosan derivatives.

As with other chitosan derivatives, the path and structure of chitosan phosphorylation products depend on the nature of the phosphorylating agent, the reactant ratio, and the reaction conditions (Figure 18) [117,118].

Usually, chitosan is phosphorylated in a reaction with solutions of phosphoric acid, triethyl phosphate, and phosphorus pentoxide. Under these conditions, low phosphorylation derivatives result that are either free or bound to chitosan by inter- and intramolecular bridges [118]. A reactant that facilitates monophosphorylation and prevents the formation of intra and intermolecular bonds is phosphorus oxychloride (POCl3). Thus, to obtain selective monophosphorylated chitosan, the polymer reacts with phosphorus oxychloride in dimethylformamide [112]. In this way, the solubility in water increases while avoiding the formation of polyphosphate. The use of POCl_3_ leads to negligible degradation of the product by breaking the O-glycosidic bonds.

To obtain N-mono- and di-phosphonic methylene chitosans that are water soluble, chitosan is treated with phosphorous acid and formaldehyde simultaneously in aqueous acidic medium [112]. A reductive amino reaction takes place, and a hydrophobic alkyl chain is introduced to the free –NH_2_ groups of N-methylene phosphonic chitosan. The result is an amphiphilic chitosan derivative that can be used like a surfactant [119]. 

### 1.12. The Complexes with Metal Ions Contribution to the Antibacterial Effect of Chitosan

Although it is unanimously accepted that the active center for the coordination of transition metal ions is the nitrogen atom of the amino group in chitosan, over time, several authors have investigated the possibility of participating in the formation of complexes and other functional fragments of chitosan, such as hydroxyl groups (especially those in the C3 position) [85] or the oxygen atom of the glycosidic bond [120].

The addition of a metal to chitosan increases its antimicrobial activity compared to native chitosan [121]. For example, the antimicrobial activity of chitosan-Zn^2+^ or chitosan-Ag^+^ is higher than that of native chitosan [122]. Loading of Cu^2+^ and Mn^2+^ to chitosan increases its antibacterial activity against *Staphylococcus aureus*, *E. coli*, and *Salmonella enteric* [82,84]. Chitosan-based silver nanoparticles show higher antibacterial activity against *Bacillus subtilis, E. coli*, and *Staphylococcus aureus* than chitosan alone [54,123].

To explain these results, several types of reactions have been proposed as possible mechanisms: adsorption, ion exchange, or formation of chelates with various metal ions. However, the type of interaction depends on the composition of the solution, the pH of the solution, and the type of metal ions. 

#### 1.12.1. The Case of Adsorption

Rinaudo established that the order of the chitosan affinity to bivalent cation adsorbed is Cu^2+^>>Hg^2+^>Zn^2+^>Cd^2+^>Ni^2+^>Co^2+^~Ca^2+^ [124]. Based on the results of the same researcher, the order of affinity to trivalent cations is Eur^3+^>Nd^3+^>Cr^3+^~Pr^3+^ [124].

Analyzing the series of mentioned trivalent metals, it is observed that the selectivity of chitosan for chromium is similar to that of praseodymium. However, praseodymium is slightly heavier than chromium, and the ionic radius of the two ions is very different (Pr^3+^, 1.013 A and Cr ^3+^, 0.63 A). From here, it can be assumed that the size of metal ions does not seem to matter so much, even if their hardness plays an important role in the selectivity of the involved ion [124]. That is why it is assumed that the kinetics of the adsorption process play an important role.

Rinaudo based his explanation of this mechanism onto spectrophotometric and potentiometric analyzes, thus proving that the affinity for the adsorption of metal ions does not depend on the size and hardness of the ions.

#### 1.12.2. The Case of Ion Exchanges

Depending on the strength of the ionic strength present in the chitosan-Me^n+^ bond, the chitosan inhibitory activity may be altered [40,69,70]. This can be explained by the attenuation of the chelating capacity, with the addition of metal ions, especially in the case of bivalent ions that are usually presents in bacteria cells (Ca^2+^, Mg^2+^) [69]. In the case of Zn^2+^ it was demonstrated that the addition of this ions inhibited the antibacterial activity of chitosan compared to Ba^2+^, Ca^2+^, or Mg^2+^ ions [125]. Chitosan expresses a polycationic character; in the environment, there are several cations with the capacity to interact competitively to bind to the negative components of the bacterial cell wall, thus weakening the antimicrobial activity of chitosan [69]. Competitiveness may also exist between environmental anions and polycationic chitosan. For example, in the case of phosphate ions, they can interact electrostatically with chitosan, on which Ca^2+^, Ba^2+^, Mn^2+^, Ni^2+^, and Cu^2+^ ions have been adsorbed [121,126].

Krajewska prepared chitosan gel membranes and extensively characterized their diffusion properties. The permeability of metal ions through these membranes was measured, and the permeability scale was as follows: Cu < Ni < Zn < Mn < Pb <Co < Cd < Ag [127]. Krajewska correlated this scale to that of chitosan–metal affinity and concluded that the diffusive properties of chitosan membranes towards metal ions offer potential for protection of chitosan-based biological systems against the destructive effects of heavy metals. This should also be extended to applications in the packaging industry [85].

#### 1.12.3. The Case of Chelates with Metal Ions

**Figure 19:**
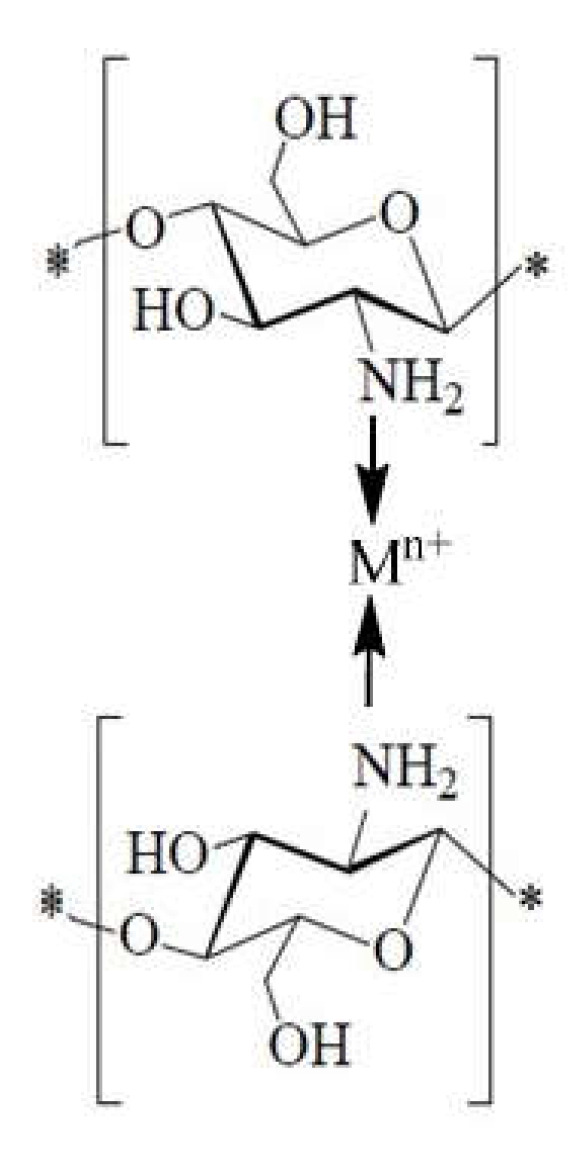
Schematic “bridge” model represented according to [112].

**Figure 20:**
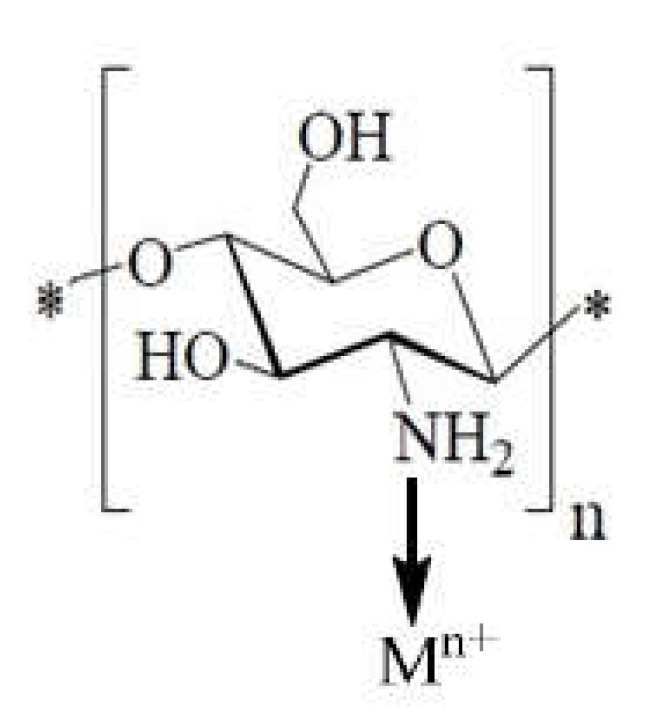
Schematic “pendant” model represented according to [112].

Over time, two types of chelating models were proposed:

1. The “bridge” model (Figure 19), involving the coordination of metal ions with several amino groups belonging to different glucosamine units in the same or different polymer chain [112].

2. The “pendant” model (Figure 20), in which the metal ion is coordinated with a single amino group [112]

Depending on the molar ratio of metal ion/ligand, the coordination of some ions can take place according to the “bridge” model or to the “pendant” model, or it is possible to form a ring with different number of sides, in which the hydroxyl group from position C-3 also participates.

However, for some ions, such as Zn^2+^ and Mn^2+^ ions, coordination is possible only with the participation of amino groups, either according to the “bridge” model or to the “pendant” model [112,128]. Similar, Ag ions can be used to obtain chitosan-Ag nanocomposites, which present remarkable antibacterial activity [129,130,131,132].

Amino groups in chitosan molecules are responsible for the absorption of metal cations by chelation. Generally, such a mechanism is more effective at a high pH value, because the amino groups are unprotected, and the electron pair in the amino nitrogen is available for donation into metal ions. Thus, it is certain that the proportion of sites available for the interaction of the chitosan skeleton with metal ions is pH-dependent [85]. At pH 6 the complexation involves only one –NH_2_ group and three molecules of hydroxyls or H_2_O, while at pH > pKa, it is likely that two –NH_2_ groups are involved in the formation of the complex. For higher pHs, i.e., 7–9, it is considered that the deprotonation of hydroxyl groups occurs, and the predominant complexation is governed by two groups: –NH_2_ and two dissociated hydroxyl groups [81]. 

**Figure 21:**
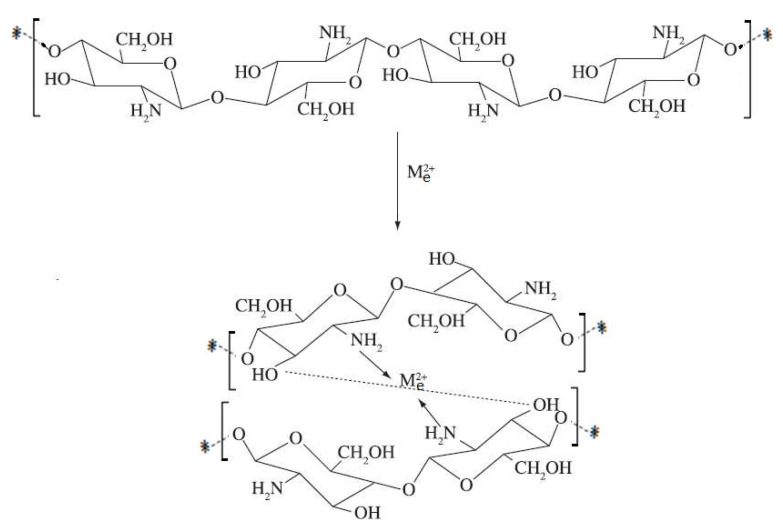
Schematic chitosan chelation with metal ions, according to [81].

Based on these arguments, a model of chitosan with metal ions complexation was proposed (Figure 21). The metal was arranged as an electron acceptor connected to one or more chitosan chains by –NH_2_ and by forming bridges to hydroxyl groups [81].

The structure of chitosan–metal complexes depend on the chitosan/metal ion molar ratio [120], type of metal ion, molecular weight of chitosan, degree of deacetylation of chitosan, zeta potential [133] of the loaded ion nanoparticles, as well as on the working conditions [74,81,87].

**In acidic conditions**, chitosan possesses high chelating capacity for various metal ions (i.e., Ni^2+^, Zn^2+^, Co^2+^, Fe^2+^, Mg^2+^, and Cu^2+^) [9,85,121,134]. For this reason, it has been widely applied for the removal or recovery of metal ions in different industries [53,86,122]. Metal ions combined with cell wall molecules of microorganisms are crucial for the stability of the cell wall. The interaction between chitosan and divalent ions that are in the microorganism cell wall prevents the production of toxins and inhibits bacterial growth. This is one of the explanations of a possible mode of antimicrobial action of chitosan [43,55,135]. Another study has shown that, by chelation, chitosan is also able to combine divalent metal ions **in neutral conditions** and exerts strong antimicrobial activity [69].

Chitosan has been known to be used as biodegradable film with good microbial resistance for food packaging. The antibacterial properties of chitosan are obtained from its polymeric structure, which has a positively charged amine group. Because of the electrostatic attraction between the polycationic chitosan and the polyanionic polysaccharides, it is possible to form polyelectrolyte complexes. Generally, it has been found that polyelectrolyte complexes (PEC) have better physico-mechanical properties than its constituent polymers due to its tighter microstructure [136].

PEC have the ability to be used in matrices, beads, microparticle and nanoparticle systems, and microspheres and can be used as film coating [136,137], being used in drug delivery systems.

The synthesizing of metal nanoparticles (e.g., AgNPs) in a chitosan matrix to form nano-biocomposites for biomedical applications is one of the achievements of the last decade [130,131,132].

In addition, a silica phase can be incorporated into the chitosan matrix to improve the mechanical properties of this biopolymer. In this way, nanofibers can be obtained that are useful in practice, because they have many advantages, including large and active surfaces that are desirable for use in sensors and as affinity membranes (e.g., air filtration membranes, water filtration membranes, scaffolds for tissue engineering) [138,139,140]. Another mechanism that can be used to overcome the poor mechanical strength of chitosan is represented by its immobilization onto the surface of other polymer supports, such as polypropylene, polyethylene, polystyrene, etc. [137,141,142,143,144].

## 2. Conclusions

This basic biopolymer, chitosan, and its derivatives that can be obtained, have a number of applications in various areas of interest. These compounds can be used in agriculture, product preservation, heavy metal ion recovery from wastewater, etc. Other specific applications can be in biomedicine and pharmaceutical fields, with remarkable results in tissue engineering and in controlled drug delivery. The area of applications may remain open to new horizons. From the perspective of the efficiency of chitosan and its derivatives as antibacterial agents, the bacteriostatic/bactericidal role is clearly proven both for non-functionalized chitosan and for chitosan derivatives in different ways.

Unfortunately, due to the wide variety of chitosan sources (from fungi, insects, crustaceans), its intrinsic properties are very varied, which creates difficulties to establish the parameters that affect the antibacterial action of chitosan and its derivatives. Therefore, the properties of the basic product, chitosan, must always be well established, so that the chosen path can be justified in order to obtain chitosan derivatives in accordance with the intended purpose.

By synthesizing the main physical–chemical properties of chitosan, its biological properties can be emphasized.

✓ Being a cationic polyamine, it is a biocompatible product.
✓ When it is positively charged, when the molecule is protonated, it adheres to negatively charged surfaces, so it displays its bioadhesion property.
✓ Due to the possibility of chitosan to form salts with organic and inorganic acids, it is a biodegradable product.
✓ Chitosan, because of its positively charged amino groups and its high charge density in acidic conditions, is able to interact spontaneously with anionic polymers to form a polyelectrolyte complex. The more monomer units there are, the higher the molecular weight of chitosan and the greater its potential to form polyelectrolytes complexes. Because the polyelectrolyte complex is generally obtained by self-assembly of oppositely charged polymers in aqueous medium without using organic solvents or toxic cross-linkers, it is considered to be safe and nontoxic.
✓ Due to the possibility of having a different degree of viscosity, it is a hemostatic, bacteriostatic, and fungistatic compound.
✓ The property of forming chelates with various metal ions makes it a suitable compound for wastewater treatment and in applications that require antibacterial effects.

The antibacterial action of chitosan will always be dependent on the intrinsic parameters that determine the properties of chitosan but will also depend on working conditions, especially on the pH of the environment, and the substrate on which it acts, the interdependence between chitosan structure and its antibacterial activity being obvious.

## Data Availability

No data were used in present review.
